# Sedimentary seagrass carbon stock and sources of organic carbon across contrasting seagrass meadows in Indonesia

**DOI:** 10.1007/s11356-023-29257-3

**Published:** 2023-08-19

**Authors:** Yusmiana P. Rahayu, Mariska A. Kusumaningtyas, August Daulat, Agustin Rustam, Devi D. Suryono, Hadiwijaya L. Salim, Restu N. A. Ati, Nasir Sudirman, Terry L. Kepel, Andreas A. Hutahaean, Novi S. Adi

**Affiliations:** 1 School of Biological Sciences and Oceans Institute, The University of Western Australia, 35 Stirling Highway, Perth, WA 6009 Australia; 2 Research Center for Conservation of Marine and Inland Water Resources, National Research and Innovation Agency, The Republic of Indonesia, Soekarno Science and Technology Area, Jl. Raya Bogor Km 46, Cibinong, Bogor, 16911 Indonesia; 3 Research Center for Oceanography, National Research and Innovation Agency, The Republic of Indonesia, Ancol Science Area, Jl. Pasir Putih I, Ancol, Jakarta, 14430 Indonesia; 4 Coordinating Ministry for Maritime Affairs and Investment Republic of Indonesia, Jl. M.H. Thamrin No. 8, Central Jakarta, 10340 Indonesia; 5 Ministry of Marine Affairs and Fisheries, The Republic of Indonesia, Gedung Mina Bahari III, Lt.11, Jl. Medan Merdeka Timur No. 16, Central Jakarta, 10340 Indonesia

**Keywords:** Blue carbon, Isotopes, Organic carbon, Climate change, MPA, Conservation

## Abstract

Seagrass meadows are an important component of coastal ecosystems globally, and they capture and store organic carbon in living biomass and sediments. Geographical estimates of blue carbon in seagrass habitats are regionally biased, with limited information from the Indo-Pacific region, including Indonesia. Seagrass extent in Indonesia is declining rapidly, and it has been suggested that marine protected areas (MPAs) are an important instrument to support protection of seagrass ecosystems and their services. Thus, this study is aimed at quantifying and comparing sedimentary carbon stocks and sources of organic carbon from seagrass meadows located in undisturbed areas outside MPA, disturbed areas outside MPA, and within MPA in three small islands in Indonesia. The sediment carbon stocks from this study ranged from 19.81 to 117.49 Mg C ha^−1^, with the highest stock measured inside MPA (77.15 ± 1.38 Mg C ha^−1^), followed by undisturbed outside MPA (36.08 Mg C ha^−1^), and the lowest stock at disturbed outside MPA (21.86 ± 0.31 Mg C ha^−1^). The predominant source of organic carbon in disturbed meadows was from coastal POM (particulate organic matter, ~ 36%), while in MPA and undisturbed sites, the main source was from seagrass, with ~ 38% and ~ 60% contributions, respectively. The results of this study add more data and information on seagrass blue carbon potential from three different islands with different degrees of disturbance in Indonesia.

## Introduction

Seagrass meadows play an important role in coastal ecosystems because they provide food and habitat for many species, support global fisheries production, help maintain water quality, and stabilize sediment (Mtwana Nordlund et al., 2016; Potouroglou et al. 2017; Unsworth et al. 2018, United Nations Environment Programme 2020). Seagrass meadows also contribute approximately 50% of the total carbon burial within the ocean, despite occupying less than 1% of the sea floor (Duarte et al. 2005; McLeod et al. 2011). Seagrasses are plants that use carbon dioxide and dissolved inorganic carbon in the air and water to photosynthesize (Duarte and Cebrián 1996). Living plant biomass contains some of the carbon stored by seagrass habitats, but the soil in which they grow contains a more substantial amount (McLeod et al. 2011). Soil carbon tends to decompose very slowly because the salty, low-oxygen conditions are not conducive to decomposing bacteria. Additionally, soil tends to accumulate, resulting in deeper soil deposit over time (Vanderklift et al. 2019). Together with mangrove forests and tidal marshes, they comprise coastal blue carbon ecosystems (Nellemann and Corcoran 2009). Additionally, due to their capacity for carbon sequestration and ability to store large amounts of carbon within the ecosystem, seagrass meadows are considered as nature-based solutions for climate change mitigation (Macreadie et al. 2021; Vanderklift et al. 2022), and their protection, management, and restoration are necessary, especially as they are declining with an alarming rate in Southeast Asia (Sudo et al. 2021).

A global estimate of organic carbon (OC) storage in seagrass ecosystems is approximately 1732–21,000 Tg C (1.7–21 × 10^9^ Mg C, Macreadie et al. 2021), with the Southeast Asian region accounting for 429.11 Tg C (0.43 × 10^9^ Mg C, Stankovic et al. 2021). The OC within seagrass meadows originates from autochthonous (seagrass-derived) and allochthonous (sestonic or terrestrial) sources (Kennedy et al. 2010). Measurements of seagrass carbon stock and source of OC are relatively scarce in the tropical Indo-Pacific relative to other regions (Waycott et al. 2009; Fourqurean et al. 2012), despite having the highest seagrass diversity in the world (Short et al. 2007), particularly Southeast Asian region. Indonesia is located within this region and has over 17,000 islands with more than 90,000 km long coastline and 875,967–1,847,341 ha seagrass extent (Martha 2017; Sjafrie et al. 2018; Sui et al. 2020), offering vast potential as a blue carbon sink (Wahyudi et al. 2020).

The variability in environmental conditions of Indonesia play an important role in shaping the characteristics of seagrass ecosystems, with implications on OC stock assessments for the region and globally (Miyajima et al. 2015; Stankovic et al. 2021). Furthermore, most of the published blue carbon literature in Indonesia has focused on organic carbon within seagrass biomass (Wawo et al. 2014; Wahyudi et al. 2020; Wahyudi et al. 2022), and only few studies reported OC values in seagrass sediment (Rahayu et al. 2019; Hertyastuti et al. 2020), where almost 97% of blue carbon is stored (Stankovic et al. 2021). Stankovic et al. (2021) quantified seagrass blue carbon in Southeast Asia, including Indonesian seagrass; however, fieldwork measurement data from Indonesia was lacking, and values were estimated using models which may not reflect the actual conditions. Additionally, Alongi et al. (2016) published data for the carbon stock of Indonesia, that included seagrass ecosystems, but the sediment carbon stock values were mainly generated from bulk density estimation based on the global models developed in Fourqurean et al. (2012), with minimal data collection and field measurements.

Currently, many countries are increasingly exploring the potential and inclusion of coastal wetlands in their national greenhouse gas (GHG) accounting. The Intergovernmental Panel on Climate Change (IPCC) provides guidelines and instructions on how to calculate GHG emissions, and removals from managed and natural ecosystems, including coastal wetlands, i.e., mangroves, tidal marshes, and seagrasses (Hiraishi et al. 2014; Howard et al. 2017; Vanderklift et al. 2022), and methodologies to calculate and award carbon offset credits from activities in seagrass, are also now available (Emmer et al. 2015; Needelman et al. 2018). Including seagrass meadows into national GHG accounting provides a huge nature-based solution potential for climate change mitigation in Indonesia; however, these ecosystems continue to decline rapidly (Unsworth et al. 2018; Hernawan et al. 2021). Therefore, protecting and managing seagrass ecosystems is an important strategy to maintain the stored and sequestered carbon in their biomass and sediments and can be a beneficial strategy for reducing net GHG emissions (Vanderklift et al. 2022).

Marine protected areas (MPAs) can be a useful tool to protect and rehabilitate coastal biodiversity (Howard et al. 2017; Wahyudi et al. 2022) including seagrass ecosystems. However, data on sedimentary carbon and sources of OC in Indonesian seagrass is limited, and patterns associated with MPA protection have yet to be explored. Wahyudi et al. (2022) studied the potential for carbon offset from seagrass ecosystems within the five MPAs in Indonesia but assessed only the OC within seagrass biomass. To address these gaps in knowledge, we studied sedimentary seagrass carbon stocks and sources of OC on three small islands in Indonesia with different characteristics: a disturbed island, an MPA, and an undisturbed area outside the MPA, with the goal of comparing carbon stocks and contributions of organic carbon between various conditions of seagrass meadows.

## Methods

### Study sites

**Fig. 1:**
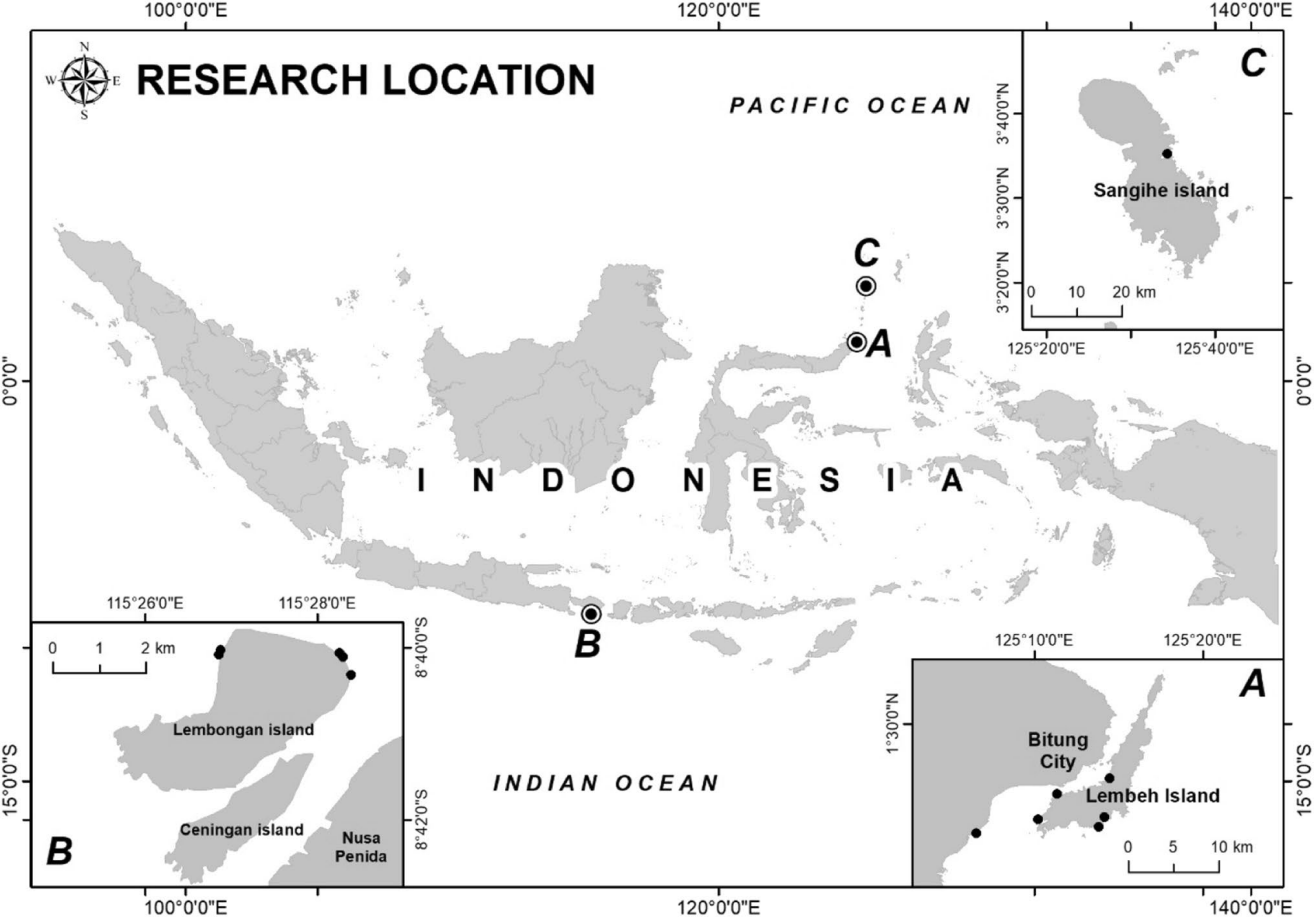
The location the study sites at Lembeh Island (**A**), Nusa Lembongan Island (**B**), and Sangihe Island (**C**). The black points represent the location of sediment cores

Three small islands (Fig. 1) were selected based on the anthropogenic setting and its influence on the seagrass meadows: Lembeh Island (a touristic island and considered as a site where seagrass is likely impacted by anthropogenic activities; hereafter, disturbed site), Nusa Lembongan Island (an MPA), and Sangihe Island (considered as a pristine, unprotected site; hereafter, undisturbed).

Lembeh is a small island in North Sulawesi Province, located in front of Bitung City, a port city with a high concentration of fishing activities (Fig. 1A). The island and the city are separated by the Lembeh Strait, characterized by small waves and steep bathymetry. The main coastal ecosystems on the island include mangrove forests, seagrass meadows, and coral reefs. Rustam et al. (2016) study estimated that seagrass cover in the island varies between 10 and 85%, and the standing biomass stock has a storage of 1.04 ± 1.3 Mg C ha^−1^ with seven seagrass species recorded: *Enhalus acoroides*, *Thalassia hemprichii*, *Halophila ovalis*, *Cymodocea serrulata*, *Cymodocea rotundata*, *Halodule uninervis*, and *Syringodium isoetifolium*.

Nusa Lembongan is an island in Nusa Penida MPA, along with Nusa Ceningan (Fig. 1B). Nusa Penida Islands is a Bali Province district declared as an MPA in 2010 and covers an area of 20,057 hectares (Carter et al. 2014). Nusa Penida MPA is an integral part of the Coral Triangle Initiative Program, where six countries (Indonesia, Malaysia, The Philippines, Solomon Islands, Papua New Guinea, and Timor Leste) committed to multilateral partnership in addressing threats to marine, coastal, and small islands ecosystems. There are three main coastal ecosystems within the MPA: mangrove forests, coral reefs, and seagrass meadows. Most of the mangrove and seagrass ecosystems can be found in Nusa Lembongan and Nusa Ceningan islands, while coral reefs surround the Nusa Penida waters. Within the MPA, 8 species of seagrass are recorded (*Halodule uninervis*, *Thalassia hemprichii*, *Halophila decipiens*, *Halophila ovalis*, *Enhalus acoroides*, *Cymodocea rotundata*, *Cymodocea serrulata*, and *Syringodium isoetifolium*) which cover an area of 108 hectares (Darma et al. 2010).

Sangihe Islands are located in the north of Sulawesi Island (Fig. 1A) and are one of the most northern groups of islands in Indonesia. The islands are bordered by Mindanao Island (The Philippines) in the north, and Sulawesi Island in the south, and are surrounded by two oceans, Sulawesi and Maluku Seas (Undap et al. 2019). Six seagrass species are recorded on this island, *Enhalus acoroides*, *Thalassia hemprichii*, *Halophila ovalis*, *Cymodocea serrulata*, *Halodule uninervis*, and *Syringodium isoetifolium* (Daulat et al. 2015).

### Sample collection

Sediment cores were collected from seagrass meadows within these three islands, using a 1-m stainless steel auger (6 cm diameter), which was manually inserted to the sediment until refusal. When the sediment was retrieved, it was sub-sectioned at the field at 5-cm intervals and frozen (− 20 C) before laboratory analyses. Ecosystem mapping was carried out before going to the field to determine the sediment core sampling point representing the distribution of seagrass at each study site. In total, 12 cores were obtained, six from the disturbed site, five from MPA, and one from the undisturbed site (Fig. 1). The initial design was to obtain a similar number of cores at each site; however, due to rough sea conditions at Sangihe Island (undisturbed), only one core was successfully secured from this site.

In the laboratory, all sediment samples were subsequently analyzed for OC content, total nitrogen (N) content, as well as stable isotope compositions (*δ*^13^C and *δ*^15^N). Prior to analysis, sediment samples were dried in an oven at a constant temperature of 60 °C, until constant weight was reached. All the samples were ground to a fine powder and acidified with hydrochloric acid to remove carbonate in the sediment. Then, OC and total N contents were obtained by flash-combustion in a CN analyzer (Eurovector EA3000 Elemental Analyzer), which was conducted at the Leibniz-Zentrum fur Marine Tropenforschung Laboratory, Bremen Germany. The sediment OC stocks were calculated using protocols and equations from Howard et al. (2014). Dry bulk density (DBD) was calculated using formula (1), then carbon density was determined by multiplying DBD with OC content (wt%) at a specific depth (formula (2)). Sediment OC stock per sampled depth interval is then calculated following formula (3). Total sediment OC stock from one core was determined by summing up all OC stock at all depth intervals from the entire core.

1 $$\textrm{DBD}=\frac{\textrm{mass}\ \textrm{of}\ \textrm{dry}\ \textrm{soil}\ \left(\textrm{g}\right)}{\textrm{original}\ \textrm{volume}\ \textrm{sampled}\ \left({\textrm{cm}}^3\right)}$$

2 $$\textrm{Carbon}\ \textrm{density}\ \left(\textrm{g}\ {\textrm{cm}}^{-3}\right)=\textrm{DBD}\ \left(\textrm{g}\ {\textrm{cm}}^{-3}\right)\times \textrm{OC}\ \textrm{content}\ \left(\textrm{wt}\%\right)$$

3 $$\textrm{OC}\ \textrm{Stock}\ \left(\textrm{Mg}\ \textrm{C}\ {\textrm{ha}}^{-1}\right)=\textrm{Carbon}\ \textrm{density}\ \left(\textrm{g}\ {\textrm{cm}}^{-3}\right)\times \textrm{depth}\ \textrm{interval}\ \left(\textrm{cm}\right)$$

Sediment OC stocks were extrapolated to 1-m depth to allow for an equal comparison to other studies. Extrapolation might cause over estimation of OC stock; therefore, the OC stock from actual depth remains presented.

Additionally, the stable isotope compositions (*δ*^13^C and *δ*^15^N) were measured using a Finnigan Deltaplus mass spectrometer coupled to a Carlo Erba Flash EA1112 Elemental Analyzer. All isotopic ratios were expressed relative to Vienna Pee Dee Belemnite for carbon, and atmospheric N_2_ for nitrogen, in per mille notation (‰). The instrument accuracy was checked by calculating the standards resulting in standard deviations as follows: C = 0.037%, *δ*^13^C = 0.069‰.

Differences in OC, DBD, total N, *δ*^13^C, and *δ*^15^N between disturbed and undisturbed sites and between MPA and undisturbed site were tested using one-sample *t*-test, because there was only one core data from the undisturbed site. Meanwhile, differences between disturbed site and MPA were tested using two-sample *t*-test with log transformation to meet the assumption of normal distribution. To estimate the proportional contribution of the different sources within the seagrass sediment, Bayesian Stable Isotope Mixing Model was used in R (simmr, Parnell and Inger 2016). The model uses the Markov Chain Monte Carlo (MCMC) algorithm to determine the proportion of source contributions while incorporating the uncertainties (e.g., isotope fractionation). Four end members as potential sources were used: seagrass biomass, mangrove detritus, terrestrial plants, and coastal particulate organic matter (POM). The reference values for isotopic signatures for mangrove detritus were from Bintuni Bay, West Papua (Sasmito et al. 2020), seagrass biomass from Spermonde Islands (Rahayu et al. 2019), terrestrial plants from Kuramoto and Minagawa (2001), and coastal POM from Jennerjahn et al. (2004). All analysis was performed in R software (R Core Team 2022).

## Results

### Seagrass sediment properties at disturbed site, MPA, and undisturbed site

Seagrass sediment properties (OC, atomic CN ratio, *δ*^13^C, and *δ*^15^N) were not significantly different between the study sites (*t*-test, all *p* > 0.05, Table 1). The OC content (%) across all three sites varied from 0.40 to 4.97%, and the highest mean was at MPA, followed by disturbed and undisturbed site (Table 1). On the other hand, DBD varied between 0.41 and 0.70 g cm^−3^ and had the opposite trend, with the highest mean value recorded at undisturbed site, followed by MPA and disturbed site (Table 1). There was a significant difference in DBD between disturbed and undisturbed sites (*t*-test, *p* = 0.004). Total nitrogen content ranged from 0.009 to 0.27% with the highest mean at MPA and the lowest at disturbed site (Table 1). There was a significant difference in total nitrogen content between disturbed and undisturbed sites and also between disturbed and MPA sites (*t*-test, *p* = 0.02 and *p* = 0.01, respectively). The *δ*^13^C values from all sediment cores ranged from − 27.36 to − 13.62‰, with the highest mean at undisturbed site and the lowest at disturbed site (Table 1). The *δ*^15^N values ranged from 1.65 to 5.49‰, with the highest mean at disturbed site and the lowest at undisturbed site (Table 1).

**Table 1:** Mean values of OC, bulk density, total nitrogen, atomic CN ratio, *δ*^13^C, and *δ*^15^N in the study sites ± SE

Site	Core ID	OC (%)	Bulk density (g cm^−3^)	N (%)	Atomic C:N ratio	*δ*^13^C (‰)	*δ*^15^N (‰)
Disturbed (Lembeh)	L01	0.74 ± 0.00	0.41 ± 0.00	0.03 ± 0.00	28.75 ± 0.86	− 26.90 ± 0.08	3.45 ± 0.05
	L02	0.52 ± 0.04	0.42 ± 0.00	0.04 ± 0.00	14.19 ± 0.27	− 18.73 ± 0.22	4.48 ± 0.07
	L03	0.42 ± 0.07	0.41 ± 0.00	0.02 ± 0.00	21.53 ± 1.04	− 21.41 ± 0.38	4.16 ± 0.20
	L04	0.44 ± 0.02	0.41 ± 0.00	0.03 ± 0.00	15.97 ± 1.15	− 20.32 ± 0.51	4.11 ± 0.16
	L05	0.28 ± 0.00	0.69 ± 0.00	0.03 ± 0.00	9.88 ± 0.24	− 13.81 ± 0.13	4.26 ± 0.16
	L06	0.61 ± 0.05	0.41 ± 0.00	0.05 ± 0.00	14.63 ± 0.13	− 18.01 ± 0.34	2.51 ± 0.04
	Mean ± SE	0.50 ± 0.07	0.46 ± 0.05	0.03 ± 0.3	17.49 ± 2.72	− 19.86 ± 1.77	3.83 ± 0.30
MPA (Nusa Lembongan)	N01	0.31 ± 0.02	0.69 ± 0.00	0.04 ± 0.00	9.31 ± 0.30	− 14.39 ± 0.15	4.51 ± 0.18
	N02	0.47 ± 0.03	0.67 ± 0.02	0.05 ± 0.00	10.16 ± 0.50	− 16.05 ± 0.22	4.40 ± 0.14
	N03	2.38 ± 0.19	0.41 ± 0.00	0.15 ± 0.01	18.96 ± 0.97	− 21.55 ± 0.49	1.98 ± 0.04
	N04	1.73 ± 0.14	0.69 ± 0.00	0.14 ± 0.01	13.97 ± 0.32	− 19.11 ± 0.27	2.82 ± 0.08
	N05	3.34 ± 0.50	0.48 ± 0.05	0.20 ± 0.02	19.55 ± 1.79	− 21.57 ± 0.57	2.53 ± 0.14
	Mean ± SE	1.64 ± 0.57	0.59 ± 0.06	0.12 ± 0.03	14.39 ± 2.14	− 18.53 ± 1.45	3.25 ± 0.51
Undisturbed (Sangihe)	S01	0.49 ± 0.02	0.69 ± 0.00	0.06 ± 0.01	9.13 ± 0.20	− 14.29 ± 0.51	2.37 ± 0.37

### Carbon stock in seagrass ecosystems

**Fig. 2:**
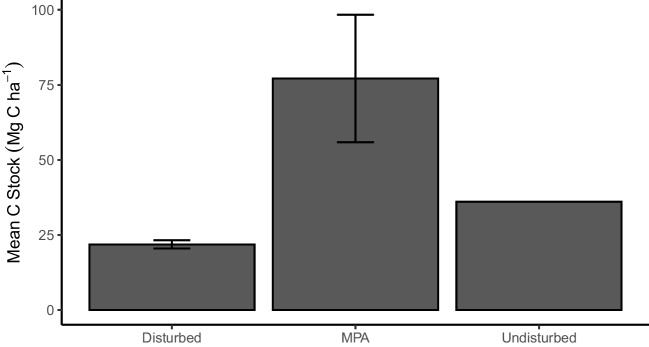
The mean carbon stock ± SE (Mg C ha^−1^) from disturbed site/Lembeh, MPA/Nusa Lembongan, and undisturbed site/Sangihe

Seagrass sediment carbon stock in the study sites varied from 3.78 to 58.18 Mg C ha^−1^ (average depth = 37 cm, *n* = 12), and when extrapolated to 1-m depth, it ranged from 19.28 to 117.40 Mg C ha^−1^. The average extrapolated seagrass sediment carbon stock was the highest at MPA, with the value of 77.15 ± 1.38 Mg C ha^−1^, followed by undisturbed site, with the stock of 36.08 Mg C ha^−1^, and the lowest was at disturbed site, with the value of 21.86 ± 0.31 Mg C ha^−1^ (Fig. 2). Significant differences of extrapolated C stock were found between disturbed site and undisturbed, also between disturbed site and MPA (*t*-test, *p* = 0.000015 and *p* = 0.02, respectively, Table 2).

**Table 2:** Summary of statistical analysis from one sample and two-sample *t*-test of sedimentary carbon stock within three study sites

Site	Sediment carbon stock (Mg C ha^−1^)	Sediment carbon stock extrapolated to 1 m (Mg C ha^−1^)
Disturbed (*n* = 6)	8.74 ± 0.42	21.86 ± 0.31^a^
MPA (*n* = 5)	32.69 ± 0.95	77.15 ± 1.38^b^
Undisturbed (*n* = 1)	10.08	36.08^b^

Superscript letters indicate significant difference between the values (*p* < 0.05)

### Sources of sedimentary organic carbon

**Fig. 3:**
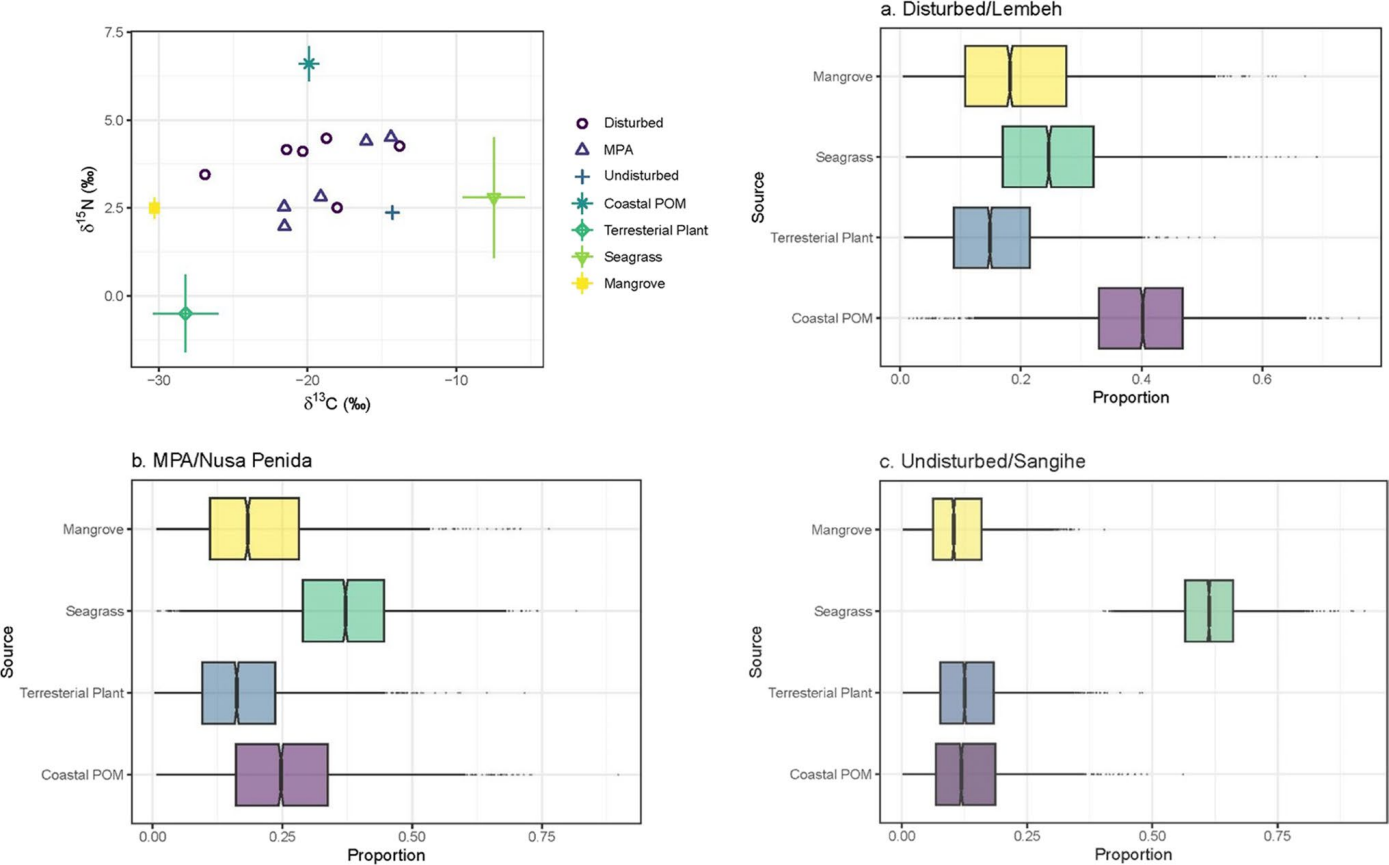
Sources and contribution of OC at disturbed site (**a**), MPA (**b**), and undisturbed site (**c**)

According to Stable Isotope Mixing Models using *δ*^13^C and *δ*^15^N (Fig. 3), the predominant source of OC at disturbed site was from coastal POM (40%), followed by seagrass (24.7%) and mangrove (19.7%; Fig. 3a). On the other hand, the main source of OC within the sediments at the MPA and undisturbed site was from seagrass, with 36.4% and 61.8% contribution, respectively. Another dominant source of carbon within sediments at the MPA was coastal POM (25.8%; Fig. 3b), but at the undisturbed site, coastal POM, mangroves, and terrestrial plants had similar contributions (13.3, 11.7, and 13.3%, respectively; Fig. 3c).

## Discussion

### Carbon stock and carbon contribution in seagrass ecosystems

The results of this study highlighted the variation of sedimentary seagrass carbon stock among three small islands in Indonesia with different intensities of human use and protection. The results suggest much lower OC stocks than national, regional, and global compilations. Based on the global estimates (194.2 ± 20.2 Mg C ha^−1^; Fourqurean et al. 2012), the OC stock is between 2.5 and almost 9 times lower at these sites and up to 1.5 times lower than the estimate for Southeast Asia (118.72 Mg C ha^−1^; Stankovic et al. 2021). Additionally, the OC stock measurements obtained from this study are 1.6 times lower than the estimates of the Indonesian seagrasses using extrapolation from surface sediment (129.9 ± 9.6 Mg C ha^−1^; Alongi et al. 2016). However, a modeled national carbon stock estimate (Stankovic et al. 2021) suggested slightly higher values than reported in this study (Table 3). The OC stock from MPA site (Nusa Lembongan) falls within the lower range of the reported OC stock in other locations in Indonesia (Table 3). However, OC stock at disturbed site (Lembeh) and undisturbed site (Sangihe) is much lower than from any reported site in Indonesia (Table 3).

**Table 3:** Sedimentary seagrass carbon stock data in Indonesia (1-m extrapolation)

Location	Min Corg stock (Mg C ha^−1^)	Max Corg stock (Mg C ha^−1^)	Mean Corg stock (Mg C ha^−1^)	Reference
National (Indonesia)	61.38	104.80	97.60 (biomass + sediment)	Stankovic et al. (2021)
Spermonde (*n* = 11)	40.54	71.08	59.3	Rahayu et al. (2019)
Riau Islands (*n* = 6)	91	103.8	97.4	Hertyastuti et al. (2020)
Lembeh (*n* = 6)	19.83	20.61	21.86	This study
Nusa Lembongan (*n* = 5)	19.28	117.4	77.15	This study
Sangihe (*n* = 1)	36.08	36.08	36.08	This study

Among the study sites, Nusa Lembongan as an MPA had the highest sediment carbon stock and OC content compared to the other two locations. The high OC content and carbon stock at the MPA site might be attributed to the composition of seagrass in the location, which is dominated by large-leaved seagrass, *Enhalus acoroides* (56.7%; Daulat et al. 2015). *Enhalus acoroides* is known as persistent species in Indonesia with high durability against environmental pressures (Risandi et al. 2023). Large-leaved seagrasses tend to have rhizomes and roots that are also larger and more persistent, penetrating deeper into sediment, making them more likely to be preserved in marine sediment (Lavery et al. 2013; Trevathan-Tackett et al. 2015; Stankovic et al., 2018; Kennedy et al. 2022). This is confirmed by Stable Isotope Mixing Model (Fig. 3b), which suggests that the main source of sediment OC in MPA site comes mostly from seagrasses (~ 36%). Seagrass meadows in this site were also located near mangrove areas (Daulat et al. 2018), which likely supply organic material to the meadows and thus increase the OC stock in the sediment, as contribution to the OC from mangrove forests was about 20.5%.

Species composition tends to affect the sediment carbon stock at the MPA site; however, this is not the case at the disturbed site. Although seagrass *Enhalus acoroides* is also dominant within the meadows (Daulat et al. 2015), sediment carbon stock at the disturbed site was lower than at the MPA site. According to the Stable Isotope Mixing Model, predominant source of OC in seagrass sediment in the disturbed site was from coastal POM (~ 36%), while seagrass contributed only ~ 26%. The lower carbon stock and contribution from the various sources within this seagrass meadow could be due to various factors. Anthropogenic disturbance such as water pollution and sedimentation from the settlement areas and port activities might affect sediment capacity to retain seagrass-derived organic matter as a similar trend was observed in the meadows in Malaysia which are highly disturbed (Rozaimi et al. 2017). Additionally, material discharge from the nearby Bitung port most likely accelerated plankton growth, which deposited within seagrass sediment when they died (Rumengan et al. 2008; Rumengan et al. 2011). Anthropogenic discharge might also have diluted the autochthonous sediment OC and caused a lower sediment carbon stock in Lembeh compared to the other two study sites. Reduction in cover, shoot density, biomass, and ultimately, OC have been observed in seagrass meadows as a result of disturbances which mainly caused by decreased water quality, increased turbidity, and eutrophication (Mazarrasa et al. 2018; Thomsen et al. 2020; Reyes et al. 2022).

Meanwhile, the lower sediment carbon stock at the undisturbed site was likely due to exposure to hydrodynamic energy. We obtained only one core in this location due to access to the seagrass meadow hindered by high waves. High wave energy environment could lower sediment deposition in the core location (Potouroglou et al. 2017; Guerra-Vargas et al. 2020), reduce seagrass photosynthesis efficiency due to increase bottom sediment resuspension, and might expose the already buried OC to aerobic condition (Mazarrasa et al. 2018, Risandi et al. 2023). However, the OC in the sediment samples from this site was mostly seagrass derived (~ 60%), which was likely due to condition of the island, since it is considered pristine with little disturbance from humans and receives little or no river discharge, therefore receiving little allochthonous input. Nevertheless, with a small sample size (only one sediment core), caution must be applied as the findings might not describe the overall condition of the study site.

### Marine protected area as a conservation of blue carbon ecosystems

The potential of MPAs to support carbon sequestration has recently been recognized (Howard et al. 2017; Reyes et al. 2022), and the results of our study also provide the comparison of seagrass sediment OC inside and outside MPA in Indonesia. In a similar study in The Philippines, the seagrass meadows within MPA had higher level of sedimentary OC compared to the disturbed meadows (Reyes et al. 2022). On the other hand, high disturbances within the seagrass meadows reduce carbon storage capacity (Rozaimi et al. 2017; Reyes et al. 2022), as seen in Lembeh Island (this study).

MPAs aim to clearly define geographical space to achieve a long-term conservation with associated ecosystem services and cultural values (IUCN WCPA 2019). There are three general zonation in Indonesia’s MPA, i.e., core, supporting, and limited used zones (Ministry of Marine Affairs and Fisheries 2020). Nusa Lembongan is a part of Nusa Penida MPA that has seven management zones, which are sustainable fishery, core, marine tourism, seaweed farming, sacred, harbor, and special tourism zone (sunfish and manta ray) (Weeks et al. 2014). Anthropogenic activities are very limited in the area, as compared to that of disturbed site, that potentially offer indirect protection to seagrass ecosystem within the MPA. Nusa Penida MPA is managed by a multistakeholder management (from community to government) that implements a marine tourism code of conduct to reduce negative impact of marine tourism on the environment (Weeks et al. 2014). A well-managed area is also expected to have a positive impact on marine ecosystems, including seagrass meadows.

In this study, the influence of MPA establishment was not directly measured relative to sedimentary carbon stock in seagrass ecosystems. This study is limited to assessing sedimentary seagrass carbon stock and sources of OC in seagrass ecosystems inside and outside MPA. It is important to conduct a more in-depth and thorough evaluation to identify how the establishment of an MPA affected carbon storage in seagrass ecosystems. One possible method is by comparing carbon stock data before and after establishment of MPA; however, this information might not be available in some locations. Alternatively, geochronological approach using a dating technique could be used to see changes in sedimentary carbon accumulation over time.

A recent study published the data that seagrass ecosystem condition in Indonesia has been described as moderately degraded (Hernawan et al. 2021). Various anthropogenic activities such as pollution, overfishing, garbage dumping, coastal development, and aquaculture are causing seagrass decline in Indonesia (Unsworth et al. 2018). Degradation of seagrass might release the carbon buried within the sediment, as the oxidation in the sediment accelerates and remineralizes OC into carbon dioxide (Serrano et al. 2016; Arias-Ortiz et al. 2018). The CO_2_ release will enter the water column, and it will potentially enter the atmosphere (Serrano et al. 2016), although more research on this is still largely needed. Hence, conserving and restoring seagrass will potentially restore OC while preserving other valuable ecosystem services such as water quality protection, and sediment stabilization.

Emphasizing the importance of seagrass blue carbon potential within the MPAs is expected to raise awareness for seagrass protection, as the current MPA programs in Indonesia are still largely focused on coral reefs and mangroves (Rifai et al. 2022). Moreover, translating ecological benefits of MPAs into economic value would assist decision-making processes by MPA managers or other stakeholders (Davis et al. 2019). A recent study in Kaimana MPA in Indonesia suggested that each hectare of conserved mangrove carbon stock, through carbon sequestration, could potentially fund the management of 53 ha of mangrove forests (Howard et al. 2017). Another study emphasized that through the conservation of mangroves within MPAs in Indonesia, avoided loss of mangrove forest was approximately 14,100 ha, which is equivalent to 13 million metric tons of blue carbon (Miteva et al. 2015). More data are still needed for seagrass ecosystems blue carbon capacity in Indonesia. One recent study estimated the value of average carbon emission reductions from seagrass biomass in five MPAs in Indonesia (Wahyudi et al. 2022), and the results from this study of sedimentary carbon stock and sources of OC from different islands of Indonesia will significantly increase empirical data of seagrass blue carbon potential for Indonesia.

## Conclusion

This study provides more data and reveals how sedimentary carbon stock and sources of OC varied within the seagrass meadows of three small islands in Indonesia. Factors such as dominant species, hydrodynamic conditions, and anthropogenic disturbances can potentially affect carbon dynamics in seagrass ecosystems. This study also discussed how MPAs could potentially help preserve blue carbon stored within the seagrass ecosystem. The low carbon stock values in this study highlight the need for local measurements of national blue carbon inventories rather than using global averages for higher accuracy. However, the use of a small number of sediment cores in this study hindered strong comparisons between sites and may not have represented the overall capability of the seagrass ecosystems in the study sites for storing carbon. Furthermore, to gain a deeper understanding of whether MPA directly influences blue carbon storage in seagrass ecosystems, more research with more core samples and analyses is strongly advised.

## Data Availability

The datasets used and/or analyzed during the current study are available from the corresponding author on reasonable request.
